# A Study of Bone Mineral Density and Its Determinants in Type 1 Diabetes Mellitus

**DOI:** 10.1155/2013/397814

**Published:** 2013-03-31

**Authors:** Ameya Joshi, Premlata Varthakavi, Manoj Chadha, Nikhil Bhagwat

**Affiliations:** Departmnent of Endocrinology, Topiwala National Medical College & BYL Nair Hospital, Mumbai 400008, India

## Abstract

Type 1 diabetes mellitus (T1DM) has been inconsistently associated with low bone mineral density (BMD) and increased fracture risk. 86 consecutive T1DM cases and 140 unrelated age and sex matched healthy nondiabetic controls were included in the study. After history and examination, BMD and body composition were assessed by dual energy X-ray absorptiometry (DXA). Serum samples were analyzed for calcium, phosphorus, albumin, creatinine, alkaline phosphatase, 25 (OH) vitamin D3, intact parathormone (PTH) levels (both cases and controls) and HbA1c, antimicrosomal and IgA tissue transglutaminase (IgA TTG) antibodies, cortisol, follicle stimulating hormone (FSH), testosterone, sex hormone binding globulin (SHBG), tetraiodothyronine (T4), thyroid stimulating hormone (TSH), growth hormone (GH), insulin-like growth factor-1 (IGF-1), and insulin-like growth factor binding protein 3 (IGFBP3) (cases only). T1DM cases had a lower BMD as compared to controls at both total body (TB) and lumbar spine (LS) (*P* < 0.05). Patients with celiac autoimmunity (CA) had significantly, lower BMD as compared to age, sex, and body mass index (BMI) matched T1DM controls. Linear regression analysis showed that low BMD in T1DM patients was associated with poor glycaemic control, lower IGF-1 levels, less physical activity (in total population as well as in male and female subgroups), and lower body fat percentage (in females) and higher alkaline phosphatase level (in males) (*P* < 0.05).

## 1. Introduction

T1DM has been variably associated with the risk of poor BMD [1–3]. The more consistent is a definite increase in fracture risk [1, 2]. There is a relative paucity of data regarding bone health in T1DM as compared to other complications, and to the best of our knowledge, no Indian data is published till date. Apart from poor glycaemic control, associated immunological disorders and loss of other bone anabolic peptides may also adversely affect bone health [1, 3]. Identifying factors affecting bone health in T1DM can help in improving bone health. Possible factors implicated in poor bone health and increased fracture risk in T1DM are enumerated in Table 1.

The objective of this study was to evaluate the difference in BMD amongst T1DM subjects and age, sex, and BMI matched controls and also to determine the various factors that might adversely impact BMD in T1DM subjects.

## 2. Methods

The study was conducted at a large tertiary care centre hospital run by the Municipal Corporation in Mumbai, India (latitude 18°55′N longitude 72°54′E). After the Institutional Ethics Committee approval (ethics committee of the T. N. Medical College, BYL Nair Hospital, Mumbai), consecutive eligible T1DM patients, who attended the outpatient department and who gave a consent/assent for the study, were included except those with proven secondary diabetes, type 2 diabetes, tuberculosis, chronic kidney disease (creatinine clearance less than 30 mg/mL), chronic liver disease, known malignancy, and pregnancy (currently pregnant or pregnancy in the last 2 years) and those on antiepileptic medications or corticosteroids. Age, sex, height, and BMI matched healthy volunteers who consented were taken as controls. The study protocol was explained to both cases and controls before participation, and a written consent/assent was taken.

A detailed history, examination, anthropometry, and pubertal status assessment of the patients were done by a single examiner. Anthropometric variables were taken in triplicate, and the mean value was considered. Physical activity was assessed by the international physical activity questionnaire as metabolic equivalent time (MET) [4]. Sensorimotor neuropathy was assessed by 10-gram Semmes-Weinstein monofilament (pressure), 128 Hz tuning fork (vibration), pinprick and ankle jerk and autonomic neuropathy by history, postural hypotension, and heart rate variability. The patient was considered to have neuropathy if he/she had any one of the positive findings. Nephropathy was assessed by 2 separate spot urine albumin/creatinine ratios done 3 months apart. The presence of retinopathy was assessed by dilated fundus examination by an ophthalmologist. Calcium metabolism parameters (serum calcium, phosphorus, albumin, alkaline phosphatase, serum creatinine, 25 (OH) vitamin D3 (radioimmunoassay (RIA), by biosource [analytical sensitivity; AS-0.5 ng/mL) and intact PTH (by immunoradiometric assay (IRMA) by immunotech; AS < 0.2 pg/mL) were assayed in both patients and controls, while HbA1c (done by ion exchange resin chromatography (at least 2 values in the last one year were averaged)), thyroid function (free T4 estimated by RIA by immunotech; AS < 0.1 ng/mL and TSH (by IRMA immunotech; AS < 0.1 uIU/mL), antithyroid antibodies (done by microtitre particle agglutination), cortisol (by RIA immunotech; AS < 0.2 mcg/dL), celiac screen by serum IgA followed by IgA TTG in IgA sufficient cases (by enzyme-linked immunosorbent assay (ELISA) from genesis diagnostics) and IgG antigliadin in IgA deficient case (by ELISA), IGF-1 (IRMA Immunotech; AS < 2 ng/mL), IGF BP3 (IRMA Beckman; AS < 0.5 ng/mL), testosterone (RIA Immunotech; AS < 0.1 ng/mL), FSH (IRMA Immunotech; AS < 0.3 uIU/mL), and SHBG (IRMA Immunotech; AS < 0.22 nM), was done only in cases. 

Areal BMD measurements of LS (L2–L4) and TB were obtained by DXA scan, done on a single lunar prodigy machine by GE medical systems (model number DF + 14230) with daily manufacturer quality control. BMC was estimated as a product of BMD and area. Precision of individual technologist for total body and lumbar spine neck was 1% and 0.9%, respectively. In population with age less than 18 years, *z* scores were derived by adjusting the BMC for age, sex, body size, and pubertal status, as per model suggested by Warner et al. which gives predictive formulae derived using regression analysis and based on the variables of age, body size, and pubertal development for interpreting measured BMC [5]. 

11 patients with CA had a lower BMD as compared to age and sex matched controls and hence were analyzed as a separate subgroup. 

Statistical analysis was done by SPSS version 19. The mean ± standard deviation (SD) was given as descriptive statistics. Log transformations were applied to highly skewed variables. Chi-square test of independence or Fisher's exact test was used to test the distribution of discrete variables. The Mann-Whitney rank sum test was used to test the difference among groups in continuous variables at baseline. Intergroup differences in DXA were estimated using the two-way analysis of covariance (ANCOVA) in which the health status (diabetes/healthy) and sex (girl/boy) were used as factors and age, height, and weight as covariates. A *P* value less than 0.05 was considered significant. A linear regression analysis was done to assess the individual effect of demographic and laboratory variables on decreased BMD. Pearson's correlation was used to determine the correlation between 2 continuous variables.

## 3. Results

The 2 groups were comparable for age (range 12–45 years), sex, height, and body mass index. There was no difference in the body fat composition in the two groups. The mean duration of diabetes was 14.61 ± 6.64 (range 1–34 years) years, and the mean age of onset of diabetes was 11.26 ± 7.94 (range 7–23 years). Differences in demographic parameters, body composition, and calcium metabolism parameters are shown in Table 2. 

Patients with T1DM had lower BMC and BMD *z* scores as compared to age, sex, and BMI matched controls at all 3 sites. Differences in BMD *z* scores in total population, individually in males and females and their distribution across age groups in both sexes for TB and LS, are given in Tables 3 and 4, respectively. The BMC and BMD *z* scores of TB and LS and the age, height, and weight adjusted contributions of diabetes (D) and sex (S) and their interaction (D × S) are given in Table 5.

TB-BMD *z* score was lower in T1DM as compared to controls (*P* < 0.01). The difference in TB-BMD was mainly due to the difference in female population (*P* < 0.01). The difference in male population was not significant in total but was significant in the 20–30 age group. The difference was also more in the >30 years age group but did not reach statistical significance, most likely due to the lesser number of subjects in that group. The bone mineral content at TB in T1DM subjects was less than age and sex matched controls (1820 ± 459 versus 1953 ± 433; *P* < 0.05). As shown in Table 5, the difference was more in females (10 percent) as compared to males (5 percent). Diabetes was an important contributor for the difference in both bone mineral content and bone mineral density (*P* < 0.05).

BMD *z* score was lower at lumbar spine in T1DM as compared to controls (*P* < 0.01). There was a significant difference in LS-BMD *z* score amongst both male and female population (*P* < 0.05). Individual age group differences across age groups did not reach significance in female subjects as the numbers were small, while there was a significantly lesser BMD *z* score amongst the 20–30 age group male T1DM subjects as compared to controls (*P* < 0.05). This age group had the maximum number of participants in the study. BMC was less at LS in T1DM subjects as compared to controls (35.2 ± 10.4 versus 39.12 ± 10.1; *P* < 0.01). Table 5 shows that both male and female T1DM participants had nearly 10 percent lesser BMC as compared to controls. Diabetes was an important contributor for the difference in both bone mineral content and bone mineral density (*P* < 0.05).

34 patients out of 86 (39.5%) had a clinical evidence of peripheral neuropathy out of which 9 had autonomic neuropathy. There was no difference in BMD amongst patients with diabetic neuropathy and those without it (BMD *z* score for TB −1.36 ± 1.21 versus −1.23 ± 1.12; *P* = 0.61 and LS −1.39 ± 1.55 versus −1.25 ± 1.45; *P* = 0.67). 16 patients had diabetic retinopathy, and their BMD was not significantly different from the T1DM population without retinopathy (BMD *z* score for TB −1.21 ± 1.43 versus −1.32 ± 1.11; *P* = 0.73 and LS −1.46 ± 1.65 versus −1.26 ± 1.38; *P* = 0.62). Patients with creatinine clearance of less than 30 were excluded from the analysis. 

Patients with CA and T1DM (6M/5F: CA− 12.79%) had a poor BMD as compared to age, sex, and BMI matched T1DM who were negative for CA, at all the three sites and hence, CA group was analyzed separately and excluded from linear regression. The comparison of anthropometry, calcium metabolism parameters, BMC, and BMD at all the 3 sites (total body, lumbar spine, and femoral neck) between T1DM patients and age and sex matched controls is given in Table 6.

Alkaline phosphatase level was elevated in CA+ patients compared to CA−. There was no difference in calcium and phosphorus levels. The differences in anthropometry, calcium metabolism parameters, and BMD in CA+ and age and sex matched CA− T1DM patients are as shown in Table 6.

**Figure 1:**
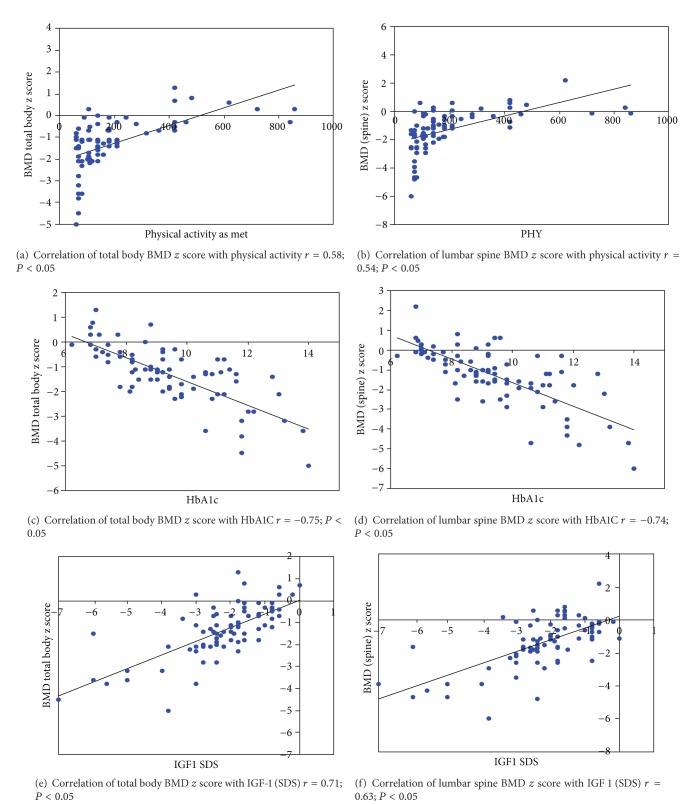
Correlation curves with coefficients of total body and lumbar spine BMD *z* score with physical activity as metabolic equivalent time (MET), glycated haemoglobin (HbA1c), and insulin-like growth factor-1 (IGF-1) standard deviation score (SDS).

**Figure 2:**
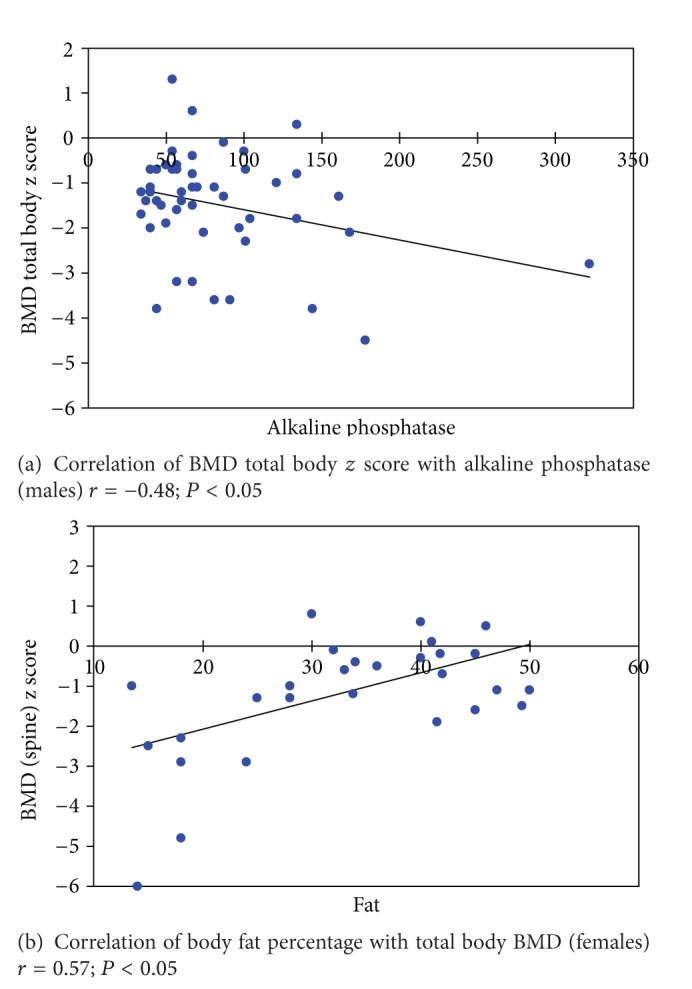
Correlation of total body BMD *z* score with alkaline phosphatase in males and with body fat percentage in females.

Linear regression analysis showed that poor BMD at TB and LS is associated with less physical activity, poor glycaemic control, and lower IGF-1 standard deviation score (SDS) (*P* < 0.05) (individual correlations of these parameters are shown in Figure 1). In addition, younger age of onset and increased alkaline phosphatase levels were associated with poor BMD for total body, while higher body fat percentage was associated with better BMD in females (individual correlation curves are shown in Figure 2) (results of liner regression analysis are as shown in Tables 7 and 8).

The number of fractures in the last 3 years was 4/11 in celiac screen-positive group, 5/75 in CA− T1DM group, and 1/140 in healthy controls (*P* < 0.05) (only cases with fracture of long bones in the lower extremities, vertebral compression fractures, or two or more long bone fractures of the upper extremities are included).

## 4. Discussion

The present study looks at the BMD differences in T1DM and age, sex, and BMI matched controls. It also tries to find out the possible factors associated with poor BMD in T1DM population. Though many studies have focused on BMD in T1DM, very few have looked at the possible determinants of poor BMD in the population. To the best of our knowledge, this is the first study in the Indian population trying to deal with BMD and its determinants in T1DM. 

The study clearly demonstrates that BMD in T1DM is low as compared to age, sex, and BMI matched controls. Previous studies regarding BMD in T1DM have been inconsistent. 3 major meta-analyses dealing with bone health in T1DM and the reports of various studies included in these meta-analyses are very variable. Vestergaard and Strotmeyer found that BMD was lower at hip and spine in T1DM and the later also found that older age and longer duration of T1DM were associated with poorer BMD [1, 3]. Vestergaard and Janghorbani found an increased risk of hip fracture in T1DM [1, 2]. No conclusive relation of glycaemic control to BMD was found in the above studies. A consistent finding observed in these studies and the 3 major meta-analyses is the increased risk of fractures [1–3]. 

Various factors have been studied, individually or in combination, by various authorities for their impact on BMD in T1DM. Factors claimed to be variably associated with poor BMD are younger age of onset of diabetes [3], female sex [1], longer duration of diabetes [3], poor glycaemic control, lower height, lower BMI, vitamin D deficiency, increased bone resorption markers, decreased bone formation, associated complications of diabetes, and associated celiac disease (CD) [1]. 

Few studies suggested increased bone resorption while others proposed loss of bone formation as the cause of low BMD in T1DM [6]. In our study, we did not test for any selective resorptive/absorptive marker except alkaline phosphatase which correlated negatively with total body BMD, but the difference is mainly due to the difference in the male population.

Reports have been variable about sex differences in bone health in T1DM. In our study, the difference in total body BMD was mainly due to the difference in female population who also had a greater deficit in BMC as compared to controls which is similar to the observations by Leger et al. In another study by Saha et al. dealing with bone mass and structure, diabetic boys seemed to be more affected than diabetic girls [6, 7]. Among the boys, the mean deficit in BMC of all measured skeletal sites was more than 10%, while among the girls it was less than 5% [7]. 

Younger age of onset of diabetes has been associated with poor BMD which continues to persist over years as noted in few studies as the one by Mastrandrea et al. [8]. In our study, the younger age of onset was associated with poor total body BMD (*P* = 0.03), but the value did not achieve significance at lumbar spine BMD (*P* = 0.09). However, we think that the value would have reached significance with more number of patients included in the study.

Anthropometry and body composition did not correlate with BMD except that females with higher fat percentage had better BMD. Higher body fat percentage has been associated with better BMD by many few studies previously, and factors like adipocytokines and increased estrogen levels have been proposed as possible reasons [8].

Better glycaemic control, a proven measure to decrease/avoid end-organ damage, also was associated with better bone health [9, 10]. Lower HbA1c was associated with better BMD. Also poor BMD was associated with lower IGF-1 levels which again is a result of poor glycaemic control [11]. Poor glycaemic control may worsen BMD and increase the fracture risk by increasing calcium excretion in urine, accumulation of advanced glycation products, inducing a proinflammatory state, causing lower IGF-1 levels (which has bone anabolic action), and lowering pH/acidosis [1, 9]. 

IGFs increase bone matrix synthesis and bone formation. Both the systemic circulating as well as the locally synthesized IGF-I contribute to bone formation. IGF-I increases osteoclastogenesis and bone remodeling. Both IGF-I and IGF-II are synthesized by bone cells and stored in the bone matrix, but IGF-1 is a more potent stimulator of osteoblastic function. PTH and PGE2 are major inducers of skeletal IGF-1 synthesis, and glucocorticoids suppress IGF-1 transcription. IGFs mediate selected effects of these hormones on bone formation [11, 12].

A previous independent study by Campos et al. reported that poor glycaemic control is associated with poor BMD, and the later also reported improvement in BMD with improved glycaemic control. However, quite a few studies done previously found no relation between BMD and glycaemic control [9].

That better physical activity is associated with better BMD; a known and time-tested fact was reemphasized in our study. A recent study by Nihlsson et al. suggests that increased physical activity is associated with the enhanced development of peak bone mass [13].

Previous studies have also looked at the prevalence of various complications and the relation with BMD. Some concluded that lower BMD was associated with the presence of retinopathy or nephropathy, while others did not [1]. An independent study by Eskildsen found poor BMD in T1DM with neuropathy [14]. In our study, we did not find any difference in BMD associated with any particular complication.

Most studies reported lower BMD in CA+ T1DM except the one by Simmons et al., where the results may be biased due to the healthy volunteer effect [15–17]. The additive BMD lowering effect of CD may be due to the underlying inflammatory state increasing bone resorption, malabsorption and increased nutritional deficiencies, associated hypogonadism, and GH resistance [16]. The lower BMI in CD may add to increased fracture risk in these patients [16].

The important limitations of the study are that bone turnover markers were not assayed. The objective evidence of neuropathy by doing nerve conduction velocity was not obtained, so a number of patients with peripheral neuropathy could have been underestimated. The HbA1c values of the last one year were used, so effects due to poor glycaemic control in past years could have been discounted. 

To conclude, poor BMD in T1DM is associated with CA, lower physical activity, poor glycaemic control, lower IGF-1 levels, higher alkaline phosphatase levels (males), and lower total body fat (females) which may translate into a higher fracture risk. Improved glycaemic control and regular physical activity may result in a better bone health apart from the other known benefits in T1DM.

## Figures and Tables

**Table 1:** Factors implicated in poor bone health and increased fracture risk in T1DM.

Factors causing low BMD	
(1) Deficiency of insulin—loss of bone anabolic action [1]	
(2) Poor glycaemic control—↑ renal calcium excretion, ↓ IGF-1/↑ IGF-BP1, acidosis, alterations in collagen, and inflammation [1, 18]	
(3) Associated deficiency of other bone anabolic peptides—for example, amylin [1]	
(4) Other associated autoimmune disorders like celiac disease [1]	
(5) Associated hypogonadism	

Factors increasing fracture risk	
(1) Hypoglycemic episodes—fits and falls [1]	
(2) Poor vision—retinopathy, cataracts, and so forth [1]	
(3) Neurologic complications—neuropathy, cerebrovascular episodes, and so forth [1]	

**Table 2:** Comparison between T1DM patients and age and sex matched controls (demography and calcium metabolism parameters) (expressed as mean ± SD).

	T1DM (*n* = 75)	Control (*n* = 140)	*P* value
Demography			
Age (years)	27.2 ± 11.2	27.2 ± 11.1	0.44
Sex (M/F)	53/22	100/40	
Anthropometry			
Height (cm)	159 ± 22	161.2 ± 21	0.49
Weight (kg)	58.7 ± 14.2	59.4 ± 16.1	0.75
BMI (kg/m^2^)	23.2 ± 8.2	23.4 ± 8.8	0.43
Body fat %	28.2 ± 11.2	30.2 ± 12.9	0.25
Calcium metabolism parameters			
Calcium corrected (mg%) (8.5–10.5)	8.67 ± 0.64	8.72 ± 0.52	0.52
Phosphorus (mg%) (3–5.5%)	3.7 ± 0.42	3.4 ± 0.37	0.88
Creatinine (mg%) (0.6–1.3)	0.9 ± 0.12	0.9 ± 0.14	0.84
Alkaline phosphatase (30–120 IU/mL)	56.3 ± 12.1	53.2 ± 11.4	0.98
25(OH) vitamin D3 (ng/mL)	41.2 ± 26.7	50.8 ± 38.4	0.08
Vitamin D deficiency (<30 ng/mL)	16 (21%)	17 (12%)	0.42

**Table 3:** Differences in bone mineral density *z* scores in T1DM subjects and age, sex, and body mass index matched controls at total body (expressed as mean ± SD).

BMD *z* score	T1DM	Control	*P* value
Total body	−1.10 ± 1.50	−0.57 ± 1.39	**<0.01**
Males (53/100)	−1.22 ± 1.05	−0.98 ± 0.88	0.08
<20 (5/8)	−1.69 ± 1.25	−1.61 ± 0.84	0.39
20–30 (37/72)	−1.51 ± 0.99	−0.79 ± 0.86	**<0.01**
>30 (11/20)	−0.65 ± 1.25	−0.23 ± 1.31	0.34
Females (22/40)	−0.86 ± 1.36	−0.01 ± 1.15	**<0.01**
<20 (5/8)	−1.45 ± 1.87	−1.14 ± 0.56	0.23
20–30 (7/12)	−1.37 ± 0.79	−0.02 ± 0.71	**0.02**
>30 (10/20)	−0.16 ± 1.05	0.58 ± 1.05	0.09

BMD: bone mineral density; T1DM: type 1 diabetes mellitus.

The numbers in brackets indicate the number of subjects in each subgroup cases and controls. For example, males (53/100) to be read as 53 T1DM cases and 100 healthy controls were in this group.

**Table 4:** Differences in bone mineral density *z* scores in T1DM subjects and age, sex, and body mass index matched controls at lumbar spine (L2–L4) (expressed as mean ± SD).

BMD *z* Score	T1DM	Control	*P* value
Lumbar spine	−1.03 ± 1.19	−0.57 ± 1.04	**<0.01**
Males (53/100)	−1.12 ± 1.37	−0.77 ± 1.05	**0.02**
<20 (5/8)	−1.84 ± 1.19	−1.68 ± 1.09	0.19
20–30 (37/72)	−1.21 ± 1.46	−0.7 ± 0.84	**0.02**
>30 (11/20)	−0.7 ± 0.77	−0.59 ± 0.82	0.33
Females (22/40)	−1.06 ± 1.77	−0.23 ± 1.38	**<0.01**
<20 (5/8)	−1.61 ± 2.36	−1.37 ± 0.98	0.21
20–30 (7/12)	−1.5 ± 2.29	0.39 ± 1.20	0.14
>30 (10/20)	−0.51 ± 0.81	−0.1 ± 1.29	0.21

BMD: bone mineral density; T1DM: type 1 diabetes mellitus.

The numbers in brackets indicate the number of subjects in each subgroup cases and controls. For example, males (53/100) to be read as 53 T1DM cases and 100 healthy controls were in this group.

**Table 5:** The bone mineral content and bone mineral density *z* scores of TB and LS and the age, height, and weight adjusted contributions of diabetes (D) and sex (S) and their interaction (D × S) (expressed as mean ± SD).

	Females		Males		*P*_D_	*P*_S_	*P*_D×S_
	T1DM	Control	T1DM	Control			
TB-BMC (gm)	1518 ± 521	1683 ± 524	1948 ± 423	2061 ± 395	**<0.01**	0.23	0.06
BMD *z* Score TB	−0.86 ± 1.36	−0.01 ± 1.15	−1.22 ± 1.05	−0.98 ± 0.88	**<0.01**	**<0.01**	0.14
LS-BMC (gm)	30.9 ± 11.5	34.6 ± 11.4	37.2 ± 9.9	41.4 ± 9.6	**0.04**	**<0.01**	0.15
BMD *z* score LS	−1.06 ± 1.77	−0.23 ± 1.38	−1.12 ± 1.37	−0.77 ± 1.05	**0.01**	**0.01**	0.38

BMC: bone mineral content; BMD: bone mineral density; TB: total body; LS: lumbar spine.

**Table 6:** Comparison of anthropometry, calcium metabolism parameters, and bone mineral density in CA-positive and negative-T1DM patients (expressed as mean ± SD).

	CA+	CA−	*P* value
Demography			
Age (years)	24.5 ± 10	25 ± 10
Gender ratio M/F	6/5	5/5
Anthropometry			
Height (cm)	150.0 ± 13.01	151.9 ± 14.34	0.71
Weight (kg)	40.36 ± 10.3	44.25 ± 14.25	0.42
Body mass index (kg/m^2^)	18.65 ± 4.72	19.02 ± 4.51	0.82
BMD “*z”* score			
Total body	−1.91 ± 1.05	−0.63 ± 0.73	**<0.01**
Lumbar spine	−1.69 ± 0.92	−0.36 ± 0.93	**<0.01**
Calcium metabolism parameters			
Calcium corrected (mg%) (8.5–10.5)	8.69 ± 0.76	8.68 ± 0.74	0.96
Phosphorus (mg%) (3–5.5%)	3.8 ± 0.82	3.4 ± 0.37	0.20
Creatinine (mg%) (0.6–1.3)	0.9 ± 0.12	0.9 ± 0.14	0.49
Alkaline phosphatase (30–120 IU/mL)	145.3 ± 86	54 ± 12.33	**<0.01**
25 (OH) vitamin D3 (ng/mL)	23.22 ± 12.09	31.2 ± 8.33	0.18
Vitamin D deficiency (<30 ng/mL)	4/11 (36%)	6/22 (27%)	0.88

BMD: bone mineral density; CA: celiac autoimmunity; T1DM: type 1 diabetes mellitus.

**Table 7:** Linear regression of parameters with bone mineral density (total body) *z* score (adjusted *R* ^2^ = 0.725; *P* < 0.01).

	*β* intercept (standardised coefficient)	*P* value
Height	0.052	0.67
Weight	0.089	0.44
Fat%	0.102	0.23
Physical activity	0.261	<0.01
Age of onset of diabetes	0.250	0.03
Duration of diabetes	0.070	0.33
HbA1c	−0.42	<0.01
Insulin dose	0.02	0.80
Calcium intake	0.05	0.62
25 (OH) vitamin D3	−0.06	0.21
Alkaline phosphatase	−0.19	0.03
PTH	−0.09	0.52
IGF-1	0.28	<0.01
Testosterone	0.13	0.12
SHBG	0.11	0.24

**Table 8:** Linear regression of parameters with BMD (lumbar spine) *z* score ( *R* ^2^ = 0.669; *P* < 0.01).

	*β* intercept (standardised coefficient)	*P *value
Height	0.094	0.48
Weight	0.003	0.98
Fat%	0.058	0.53
Physical activity	0.215	<0.01
Age of onset of diabetes	0.16	0.09
Duration of diabetes	−0.046	0.56
HbA1c	−0.451	<0.01
Insulin dose	0.20	0.07
Calcium intake	0.05	0.53
25(OH) vitamin D3	−0.003	0.964
Alkaline phosphatase	−0.19	0.09
PTH	−0.026	0.71
IGF-1	0.31	<0.01
Testosterone	0.14	0.12
SHBG	0.11	0.24
